# Lexical and Structural Cues to Discourse Processing in First and Second Language

**DOI:** 10.3389/fpsyg.2021.685491

**Published:** 2021-07-01

**Authors:** Ludivine Crible, Mathis Wetzel, Sandrine Zufferey

**Affiliations:** ^1^Institut Langage & Communication, Catholic University of Louvain, Louvain-la-Neuve, Belgium; ^2^Institut de Langue et de Littérature Françaises, University of Bern, Bern, Switzerland

**Keywords:** discourse relations, contrast, connectives, parallelism, discourse processing, first language, second language

## Abstract

Discourse connectives are lexical items like “but” and “so” that are well-known to influence the online processing of the discourse relations they convey. Yet, discourse relations like causality or contrast can also be signaled by other means than connectives, such as syntactic structures. So far, the influence of these alternative signals for discourse processing has been comparatively under-researched. In particular, their processing in a second language remains entirely unexplored. In a series of three self-paced reading experiments, we compare the reading patterns of contrastive relations by native French-speakers and non-native speakers of French with English as a first language. We focus on the effect of syntactic parallelism and how it interacts with different types of connectives. We test whether native and non-native readers equally recruit parallelism to process contrast in combination with or without a connective (Experiment 1), with a frequent vs. infrequent connective (Experiment 2) and with an ambiguous vs. unambiguous connective (Experiment 3), thus varying the explicitness and ease of retrieval of the contrast relation. Our results indicate that parallelism plays an important role for both groups of readers, but that it is a more prominent cue for non-native speakers, while its effect is modulated by task difficulty for native participants.

## Introduction

Processing a text amounts to more than understanding the content of single sentences: it also involves understanding the coherence links that connect sentences to each other and to the text as a whole. These higher-level inferences correspond to *discourse relations*, a term used here to designate the mental representations of the connection between two (or more) propositions. Discourse relations are found everywhere in a text and they can express meanings of cause, condition, elaboration or contrast, among other things (e.g., Sanders et al., 1992).

Discourse relations can be identified from the content of the propositions alone, as in Example (1), where the relation of contrast can be considered implicit. Alternatively, speakers and writers can use a connective to explicitly signal the intended coherence relations. For example, the connective *whereas* signals a relation of contrast in Example (2).

(1) John has a large house in the suburbs. Mary lives in a studio downtown.
(2) John has a large house in the suburbs whereas Mary lives in a studio downtown.

In the psycholinguistic literature, many studies have demonstrated that relations explicitly cued by a connective are processed faster than implicit relations, both for adult native speakers and younger readers (e.g., Britton et al., 1982; Murray, 1997; Sanders and Noordman, 2000; Cain and Nash, 2011). Studies on non-native readers are scarcer and tend to show that the instruction of the connective may not be as straightforward, depending on its register or semantic transparency (Zufferey and Gygax, 2017; Wetzel et al., 2020), as well as possible transfer effects from the readers' first language (Zufferey et al., 2015). However, so far, non-native discourse processing remains under-researched considering the large panel of discourse relations and connectives.

A further aspect that is only beginning to attract interest to deepen our understanding of discourse processing is the role of other types of cues from the semantic or syntactic levels, and how these cues interact with connectives. Corpus studies (Das and Taboada, 2018; Hoek et al., 2019; Crible, 2020) have shown for example that a wide range of contextual cues can contribute to the marking of discourse relations, from semantic relations to syntactic constructions and even punctuation signs. One of them is structural parallelism, which consists in repeating the same argument structure across the connected segments, as in Example (3).

(3) John has a house in the suburbs. Mary has a studio in the city.

In natural language, this type of repetition frequently occurs in contrastive relations, although it is not exclusive to contrast (e.g., subordination environments, Sturt et al., 2010). The effect of non-connective cues on processing has only recently been investigated with native speakers (e.g., Grisot and Blochowiak, 2019; Crible and Pickering, 2020; Schwab and Liu, 2020) and shows interesting interactions with the connective already present in the relation. These studies focus on single pairs of connectives (e.g., *and* vs. *but*), and comparisons across various types of connectives are still lacking. More fine-grained distinctions between connectives would thus complement previous findings by further refining the “continuum from implicit to explicit discourse marking” (Crible, 2020, p. 12) and may highlight conditions in which non-connective cues are more useful than others. Furthermore, how these interactions impact non-native readers remains entirely to uncover.

In the present paper, we report three self-paced reading experiments that address this gap by investigating how parallelism interacts with different kinds of connectives in the processing of contrastive relations by native and non-native French readers. We compare implicit vs. explicit relations (Ø vs. *par contre* “however”; Experiment 1), connectives from the spoken vs. written modes (*par contre* vs. *en revanche* “by contrast”; Experiment 2) and specific vs. ambiguous connectives (*mais* “but” vs. *et* “and”; Experiment 3).

### Processing Instructions for Discourse Relations

Discourse relations such as contrast can be expressed through an explicit connective which helps readers in identifying the intended coherence relation. Connectives reduce the processing cost of discourse relations compared to their implicit versions, as measured by shorter reading times (Sanders and Noordman, 2000) and faster and more accurate answers to comprehension questions (Millis and Just, 1994). The effect of connectives is however not equal across discourse relations. The facilitation effect of connectives seems to be particularly strong for contrastive (or “adversative”) relations, compared to additive or causal relations, which are considered to be cognitively simpler and more expected in discourse (Murray, 1995, 1997).

Processing ease can in addition vary with the type of connective that is used to express it. For instance, the frequency of a connective in language use or its restriction to formal registers can make it less accessible for readers with low exposure to print (Zufferey and Gygax, 2020) and can cause immediate processing delays (Zufferey et al., 2018). In addition, when connectives can express multiple relations (e.g., *but* in contrast or concession, *since* in temporal or causal relations), comprehenders tend to be biased toward their most frequent meaning, while the secondary use is rated lower and read more slowly (Asr and Demberg, 2020). Similarly, the connective *and* can be found in many different discourse relations in corpora (Prasad et al., 2018) and in production data (Koornneef and Sanders, 2013). Spooren (1997) described *and* as an “underspecified” connective which needs to be enriched by pragmatic inferences in order to be interpreted causally or contrastively. This is reflected in Cain and Nash's (2011) study, where they found that using *and* instead of a more specific connective such as *so* for result or *but* for contrast increased reading times, which reflects “the time spent computing the appropriate relation between two clauses, which is implied but not stated” (Cain and Nash, 2011, p. 436). In sum, connectives that are rare, polysemous or underspecified might provide suboptimal instructions for discourse processing compared to more informative options.

Such variation in the informativeness of connectives arguably applies to non-connective cues as well. For instance, Grisot and Blochowiak (2019) did not find any interaction between temporal connectives and different verb tenses, against their expectations, and conclude that verb tense alone cannot act as a cue in temporal relations. Similarly, Canestrelli et al. (2016) found no facilitation for causal connectives in subjective causal relations following subjectivity markers such as *horribly*. Other studies, however, suggest that non-connective cues can be used to predict the upcoming discourse relation. A number of studies have focused on implicit causality verbs, such as *blame* or *admire*, which trigger expectations for a causal relation, sometimes in combination with a connective (e.g., Koornneef and Sanders, 2013). Studies on relations other than causality are much scarcer. Scholman et al. (2020) focused on list signals (e.g., *a few, multiple*) and found that participants produced list continuations when prompted by the signal, and that this sensitivity was especially strong for participants with a high exposure to print. Schwab and Liu (2020) looked at the *true… but* and *zwar… aber* constructions in English and German in combination with what they call “contextual cues,” i.e., the incompatibility between two propositions (e.g., owning a treadmill yet running outdoors often). They found that the lexical cues *true* and *zwar* facilitate online processing (i.e., decreased reading times at the connective region), while contextual cues mainly improved offline measures (coherence ratings). Overall, these studies thus indicate that connectives are important markers that guide the expectations of readers while processing discourse. They also indicate that connectives, mostly restricted to basic conjunctions, seem to interact with other segment-internal indicators, but the interrelations between both types of cues, including different types of connectives, remain largely to be explored. This paper is an attempt in that direction.

### Discourse Processing in a Second Language

Research on discourse processing in second language has investigated the extent to which non-native speakers process discourse like native speakers. While some studies claim that L2 learners process discourse on a shallower level than native speakers (Clahsen and Felser, 2006), others suggest that native-like discourse processing can be attained, provided a high language proficiency in L2 (e.g., Ionin et al., 2019; Cho, 2020).

But whereas second language research has focused on the processing of morphosyntactic elements in L2 such as articles (e.g., Cho, 2020), L2 inflection (e.g., Hopp, 2010), or pronoun position (e.g., Requena and Berry, 2021), the literature on processing coherence relations and connectives in L2 is rather sparse. Among the few studies that explicitly assessed benefits and potential disadvantages of connectives for reading and processing in L2, Zufferey et al. (2015) demonstrated that advanced non-native speakers were able to intuitively detect non-native uses of connectives (“if,” “when”) at a native-like level while reading, despite negative transfer effects from their L1. However, when explicitly judging the same sentences, participants fell prey to negative transfer effects. The authors concluded that non-native speakers can “reach a native-like implicit ability to understand connectives in L2” (Zufferey et al., 2015, p.406), but that this intuitive comprehension is not always matched by a corresponding metalinguistic understanding (see also Orfitelli and Polinsky, 2017).

There is also evidence however that processing discourse in L2 can be affected, even disturbed, when the meaning of the connective is not accessible for non-native speakers. Zufferey and Gygax (2017) showed, using both online and offline tasks, that even highly advanced learners of French struggle to master the ambiguous connective “en effet” which can either introduce a confirmation relation (similar to the English “indeed”), or a causal relation (similar to the English “for”). In their online experiment, learners did not benefit from the presence of “en effet” in confirmation relations to the same extent as native speakers, as they did not have native-like differences between reading times for confirmations that were appropriately marked by this connective and (incoherent) implicit confirmations. The authors thus concluded that the polyfunctional connective remained challenging for L2 learners even at an advanced level.

In sum, while there is evidence that learners can show native-like processing patterns of discourse connectives (e.g., Zufferey et al., 2015), one can also expect that L2 learners show differing reading patterns than native speakers when they do not master the connective used to convey them (Zufferey and Gygax, 2017). In addition, less frequent connectives or connectives from a higher language register could also perturb online processing, as those factors are known to affect learners' competence with connectives in offline tasks (Wetzel et al., 2020). Finally, it is so far unknown whether non-native speakers use structural cues such as parallelism when processing discourse, as no experiment has assessed this factor. This study will fill this gap in the literature.

### Parallelism as a Cue to Contrastive Relations in L1 and L2

We established in the previous sections that elements from the segments of a discourse relation can be used as processing cues, but that research in this domain is still scarce. In this study, we focus on two main types of cues: lexical cues in the form of explicit connectives (e.g., *par contre* “however”), and structural cues in the form of verb phrase parallelism. In natural language, parallelism can occur in contrastive and additive relations but is more frequent in the former (10.86 vs. 3.62%), according to Crible's (in press) corpus study on English. This focus on parallelism is motivated by findings reported in Crible and Pickering (2020), who showed that parallelism speeds up the processing of contrastive relations for native speakers. In their study, the authors manipulate the strength of the signal given by English connectives (appropriate *but* vs. underspecified *and*) and the use of the same verb phrase or a different one across the two discourse segments, as in Examples (4) and (5) below, taken from their paper.

(4) Nick always eats in low-budget restaurants and/but Grace always eats in fancy places.
(5) Nick goes to low-budget restaurants and/but Grace always eats in fancy places.

In their first two experiments, participants performed a self-paced reading task followed by a simple verification task targeting any element in the sentence. These experiments yielded a facilitation effect of parallelism that helped processing with both *and* and *but*, and failed to show a significant interaction between the structural and the lexical cues. In the third experiment, however, they changed the task to a harder comprehension question which specifically targeted the type of discourse relation expressed in the sentence, along with a number of other changes in design. In this third experiment, they found a significant interaction, such that the effect of parallelism was stronger when combined with the connective *and* than with *but*, which may be due to the deeper understanding required to perform this task. Crible and Pickering (2020) suggest that parallelism can act as a contrastive cue that compensates for the ambiguity of *and*, thus helping processing. By contrast, the instruction of *but* is informative enough, and thus processing is not affected by the use of parallel structures. They conclude that parallelism is a robust cue across the board but that subtle differences between connectives might only emerge in “active” (as opposed to “shallow”) reading, thus pointing at a possible relation between discourse cues and cognitive demands.

In the present study, we take Crible and Pickering's (2020) findings as our starting point for the investigation of the interaction between parallelism and different types of connectives. Their results suggest that parallelism is particularly beneficial with ambiguous connectives. We can therefore expect that the same pattern will be observed in the comparison between explicit and implicit relations: the added difficulty of an implicit relation (i.e., without a lexical connective) will be compensated for by the presence of the structural cue, while the presence of an explicit connective such as *par contre* will reduce the effect of parallelism. This hypothesis will thus lead us to assess whether implicit processing differs from ambiguous processing: is no information similar to low information, or does the lexical instruction of an ambiguous connective create a different inference pattern, e.g., by blocking some interpretations (Blakemore and Carston, 1999)? Similarly, any processing disadvantage associated with low-frequency connectives is expected to disappear or be reduced in contrastive relations using parallel structures. Our results will thus complement previous studies that focused on pairs of basic connectives, which fails to reflect the actual diversity of connectives. In doing so, we hope to refine our understanding of the conditions in which parallelism helps discourse processing.

Furthermore, we extend the scope of Crible and Pickering's (2020) study to non-native readers, as it is probable that L2-readers may also rely on other cues than discourse connectives (such as parallelism) in order to process a sentence. Given that parallel structures appear to facilitate processing for native speakers (e.g., Crible and Pickering, 2020), it is highly probable that the repetition of an element will also facilitate the identification of the intended coherence relations for non-native speakers. We therefore expect to observe a faster processing for sentences containing parallel structures for L2 readers. In fact, we posit that non-native readers will benefit even more from the signaling of parallelism than native readers, given that L2 readers have to rely on all available information during the demanding task of decoding a non-native language, whereas L1 readers do not depend on these cues to the same extent because decoding is less costly and leaves more room for inferences. There are also reasons to believe that parallelism can be a more accessible cue than connectives for L2 readers, as previous studies have shown that they struggle to activate and interpret the correct procedural information when it is indicated by an unfamiliar connective (Zufferey and Gygax, 2017). When the underlying coherence relation is however indicated by a highly accessible connective (i.e., a more frequent or a “transparent connective” following Wetzel et al., 2020), L2 readers should be able to fully rely on the connective and benefit from its' presence.

Thus, while mastery of connectives can be challenging for non-native readers (cf. Wetzel et al., 2020; Zufferey and Gygax, 2020), parallelism can be expected to provide a more straightforward cue helping them to infer a relation of contrast, which is why we expect a pervasive facilitation effect of parallelism for all sentences for these readers compared to native speakers, who only use it in the absence of clear connectives. However, there is, to the best of our knowledge, no evidence for these assumptions yet.

Overall, we investigate whether structural parallelism is recruited as a contrastive cue by native and non-native readers alike, and how both populations process the interaction between structural and lexical cues of different types. This first-ever study on non-connective discourse cues in a second language will therefore further our understanding of discourse processing by native and non-native speakers alike. Specific predictions will be detailed for each experiment below.

## Experiment 1

### Predictions

In Experiment 1, we examine how the presence of an explicit connective and the use of parallel structures impact discourse processing for native (L1) and non-native (L2) readers. To do so, we will measure reading times of the final segment of the discourse relation (end of second sentence) collected through a self-paced reading task. We expect that connectives will reduce reading times compared to implicit relations, as has been repeatedly shown in previous studies. We further expect that parallelism will facilitate the processing of contrastive relations compared to non-parallel conditions, by structurally highlighting the contrast between segments. In addition, we can expect an interaction such that the effect of parallelism will be stronger in implicit relations, where it will compensate for the absence of a lexical instruction (i.e., no connective). Finally, we expect all these differences to be larger in the L2 group, for whom implicit and non-parallel relations should be particularly difficult, while L1 participants will find all conditions relatively easy to process.

### Participants

For this experiment, 80 native and 80 non-native speakers of French were recruited on the web-based crowdsourcing platform *Prolific* (55% female, 33yo on average, aged 18–69yo) and were remunerated £2.5 for their contribution. L1 participants had registered French as their mother tongue and L2 participants all had English as first language and French as one of their “fluent languages” according to the screening criteria on Prolific. All participants had satisfying ratings in previous experiments on Prolific. All participants gave their informed consent before entering the study.

### Materials

Materials include 40 pairs of sentences that expressed a contrastive relation between two discourse segments. We define contrast as a comparison relation where an attribute of the first segment is present but different in the second segment, and there is no causality to infer between them. This typically involves opposites or different members of a category, such as *morning* vs. *evening* or *gold* vs. *silver*. Contrary to concessive relations which are rarely encoded in the lexical content of the segments, contrast is mostly conveyed through semantic relations (Crible, in press), although these are not always direct antonyms, as in Example (6).

(6) Rosalie pense s'orienter dans les sciences. Par contre, Fred veut faire carrière dans la littérature.
  *Rosalie thinks about studying science. By contrast, Fred wants to pursue a career in literature*.

In this example, science and literature are contrasted as different orientation choices. We manipulated the presence of the connective *par contre* (“by contrast”) to create explicit (as in 6) and implicit conditions, as in (7). We chose *par contre* as the relation-conveying connective, since it has a high frequency in speech, contrary to the more formal *en revanche* (“however”), which is bound to the written mode (Wetzel et al., 2020). The absence of the connective in (7) makes the relation arguably harder to identify, although the contrast is still expressed through the content of the segments.

(7) Rosalie pense s'orienter dans les sciences. Fred veut faire carrière dans la littérature.

We further manipulated the structure of the materials by creating parallel conditions where the verb phrase of the first segment is repeated in the second segment, as in (8).

(8) Rosalie veut faire carrière dans les sciences. Fred veut faire carrière dans la littérature.
  *Rosalie wants to pursue a career in science. Fred wants to pursue a career in literature*.

Contrast in parallel conditions is thus conveyed syntactically as well as semantically. Using similar materials (in English), Crible and Pickering (2020) reported a robust facilitation of parallelism in contrastive relations. These manipulations result in a 2 × 2 within-participant design as presented in Table 1.

**Table 1:** Conditions for Experiment 1.

Explicit parallel	Lucas s'intéresse aux films réalistes. Par contre, Kévin s'intéresse à la science-fiction. *Lucas is interested in realistic movies. By contrast, Kévin is interested in science fiction*.
Explicit non-parallel	Lucas regarde plein de films réalistes. Par contre, Kévin s'intéresse à la science-fiction. *Lucas watches many realistic movies. By contrast, Kévin is interested in science fiction*.
Implicit parallel	Lucas s'intéresse aux films réalistes. Kévin s'intéresse à la science-fiction.
Implicit non-parallel	Lucas regarde plein de films réalistes. Kévin s'intéresse à la science-fiction.

All clauses follow the same three-component pattern: first name, verb in the present-tense, object or complement. The first segment in the parallel and non-parallel conditions have the same number of syllables, and the second segment is always the same across conditions. The four conditions of the 40 items are fully counter-balanced and no participant saw the same item twice. In addition to the stimuli, 60 fillers were created, all following the same pattern. Among the fillers, 20 items express a result relation, as in (9), 20 express a causal relation as in (10), and another 20 express a relation of similarity, as in (11).

(9) Vincent est malade de la grippe. Du coup, Karen est partie à la pharmacie.
  *Vincent is sick with the flu. As a result, Karen went to the drugstore*.
(10) Yvan est très fatigué ce matin parce que Lindsay a fait du bruit toute la nuit.
  *Yvan is very tired this morning because Lindsay made a lot of noise all night*.
(11) Camille est très active sur les réseaux sociaux et Lily aussi passe des heures sur Facebook.
  *Camille is very active on social media and Lily too spends hours on Facebook*.

A number of result and additive fillers use a parallel structure as in (12), so that parallelism would not only be found in contrast, thus making it less obvious to participants. Half of all fillers had an explicit connective (as in the examples above) while the relation was implicit in the other half (i.e., the connective was removed).

(12) Bastien est très à l'aise devant un public et Léila aussi est très à l'aise sur scène.
  *Bastien is very comfortable in front of an audience and Léila too is very comfortable on stage*.

### Pretest

We pre-tested the materials (stimuli and fillers) in order to make sure that the target relation was accessible to L1 and L2 participants alike. This allowed us to identify ambiguous or unclear items. To do so, we ran a connective insertion task which presented the materials in their implicit version. Participants (80 in each group) had four options to choose from, each of them represented one of the target relations and they were the same as those used in the main experiment. Half of the items were parallel and half were non-parallel. The pretest returns an accuracy of 84% for contrastive items (86% for the L1 group, 82% for L2). A total of 11 items (including four contrastive stimuli) had a mean accuracy below 70% in one or both groups of participants, and were replaced by clearer items.

### Procedure

All items are split into six segments, represented by forward slashes “//” in (13) below. In the implicit condition, the connective region only contains the subject of the second sentence. For parallel conditions, the repeated portion always corresponds to the middle segment of each sentence, and the final segment is always different across the first and second sentence, since this is where the contrast is semantically expressed.

(13) Rosalie // pense s'orienter // dans les sciences. // Par contre, Fred // veut faire carrière // dans la littérature.

The task uses a self-paced reading paradigm where participants first see crosses at the center of the screen as a fixation point. By pressing the space bar on their keyboard, participants were able to show the first and subsequent segments one at a time. Once all six segments were read, a comprehension question appears on screen. The question directly targets the discourse relation expressed in the item and is always phrased as follows: “*Ce que Rosalie fait est … ce que Fred fait*” (“what Rosalie does is … what Fred does”), following the task used by Crible and Pickering's (2020) third experiment. They found that this disambiguation question was adequate to observe subtle differences in discourse processing and avoided passive, shallow reading strategies compared to more general verification statements. Four options were displayed on the screen: *différent de* (“different from”), *similaire à* (“similar to”), *causé par* (“caused by”), *la raison de* (“the reason for”), which correspond to the four relations under scrutiny (contrast, similarity, cause and result, respectively). Participants were instructed to click on the option that best fits the context. Fixation crosses and the next item then appeared on screen. This comprehension question aims at ensuring the participants' active reading and will be used to discard inattentive participants.

Following this main reading task, we used a lexical proficiency test (French version of Lextale, Brysbaert, 2013) in order to provide an objective assessment of the two groups of participants. They saw 80 words one at a time on the screen and had to press “v” (for *vrai* “real”) if they thought the word existed in French or “f” (for *faux* “fake”) if they did not recognize the word. Participants were instructed that words wrongly identified as real would deduct one point from their score.

The whole experiment was not time constrained. The order of presentation of all items was individually randomized. The experiment was designed on Psychopy (Peirce et al., 2019) and run online. Participants took about 30 min to complete the study on average.

### Data Analysis

For all experiments in this study, we computed linear mixed effects regression models with the *lmer* function of the {lme4} package (Bates et al., 2015) of *R* (R Core Team, 2021), and used the *anova* function for model selection (Baayen et al., 2008). We measured *post-hoc* comparisons with the *glht* function of the {multcomp} package (Hothorn et al., 2008), with Tukey pair-wise comparisons applying Bonferonni correction. We log-transformed reading times to normality, as it is standard practice to reduce positive skewed data (e.g., McKillup, 2011), although recent papers show that they might be robust enough with large samples (e.g., Schielzeth et al., 2020). We first conducted analyses separately for the L1 and L2 data because of very large differences in response times, which prevented smaller yet significant within-group differences to emerge; models with both data further allowed us to test for the effect of language group. The pre-registration form, along with complete materials and analysis scripts for all three experiments, are available at https://osf.io/mwy7q/?view_only=57847a6801e04bdead0a51f1bab97a0f.

### Results

The 80 L1 participants reached an overall accuracy of 85.4% on the comprehension question in this experiment (range 53–96), which shows that they were reading for understanding. The 80 L2 participants reached 76.73% overall accuracy (16–96). We discarded six L2 participants who scored below 50% for contrastive items. We then removed extreme reading time values above 5,000 ms (75 cases) and under 50 ms (1 case) on the critical region (final segment). Finally, we replaced any outlier above or below 2.5 standard deviation of the participant's mean, which corresponds to 204 values (3.35% of the data).

We only report results for the final segment (segment 6), which is the critical region where the discourse relation can be identified and the only segment that is directly comparable across conditions. Starting with the L1 data, the model with random intercepts by participant and by item was significantly improved by adding Structure as predictor (Δχ^2^ = 8.94, Δ*df* = 1, *p* < 0.01) but not Connective (Δχ^2^ = 2.18, Δ*df* = 2, *p* = 0.34). The mixed effect linear regression on the final model returns a main effect of parallelism, with faster reading times in the parallel condition (*M* = 735 ms; *SD* = 431) than in non-parallel items (*M* = 767 ms; *SD* = 462). Coefficients are reported in Table 2.

**Table 2:** Model of the L1 data (Experiment 1).

	**β**	***SE***	***t***	***p***
Parallel structure	−0.03	0.01	−2.99	^**^

** *p < 0.01*.

Turning to the L2 participants, the base model with random intercept per participant and item was improved by Structure (Δχ^2^ = 11.42, Δ*df* = 1, *p* < 0.001) and by Connective (Δχ^2^ = 88.09, Δ*df* = 2, *p* < 0.001). The final model returns a marginal main effect of parallelism and a main effect of Connective (see coefficients in Table 3). Reading times are shortest in the parallel-explicit condition (*M* = 927 ms; *SD* = 561), followed by non-parallel-explicit (*M* = 949 ms; *SD* = 564), parallel-implicit (*M* = 1,029 ms; *SD* = 594), and longest in non-parallel-implicit (*M* = 1,080 ms; *SD* = 577), where the relation has no lexical or structural cues. Pairwise comparison shows that all differences between conditions are significant except that between parallel and non-parallel items for explicit relations.

**Table 3:** Model of the L2 data (Experiment 1).

	**β**	***SE***	***t***	***p***
Parallel structure	−0.04	0.02	−1.77	
Implicit connective	0.15	0.02	7.27	^***^

*** *p < 0.001*.

L1 participants had a mean score of 44.76 on the Lextale test (range 21–56, maximum score 56) against 23.69 for L2 participants (1–53), which is a highly significant difference (ß = −0.38, *SE* = 0.03, *t* = −13.85, *p* < 0.001) that confirms the participants' own assessment of nativeness.^1^ We tested for an effect of group on reading times by combining both datasets. The base model with random intercepts per participant and item was improved by Structure (Δχ^2^ = 20.22, Δ*df* = 1, *p* < 0.001), by Connective (Δχ^2^ = 62.03, Δ*df* = 2, *p* < 0.001) and by Group (Δχ^2^ = 76.53, Δ*df* = 4, *p* < 0.001). The final model returns a main effect of group with longer RTs in L2 and a significant interaction between Group and Connective, suggesting that the effect of implicit relations is only true for L2 participants (coefficients in Table 4).

**Table 4:** Model of both L1 + L2 data (Experiment 1).

	**β**	***SE***	***t***	***p***
L2 Group	0.27	0.06	4.92	^***^
Group ^*^ Connective	0.12	0.03	4.8	^***^

* *p < 0.05 and*

*** *p < 0.001*.

**Figure 1:**
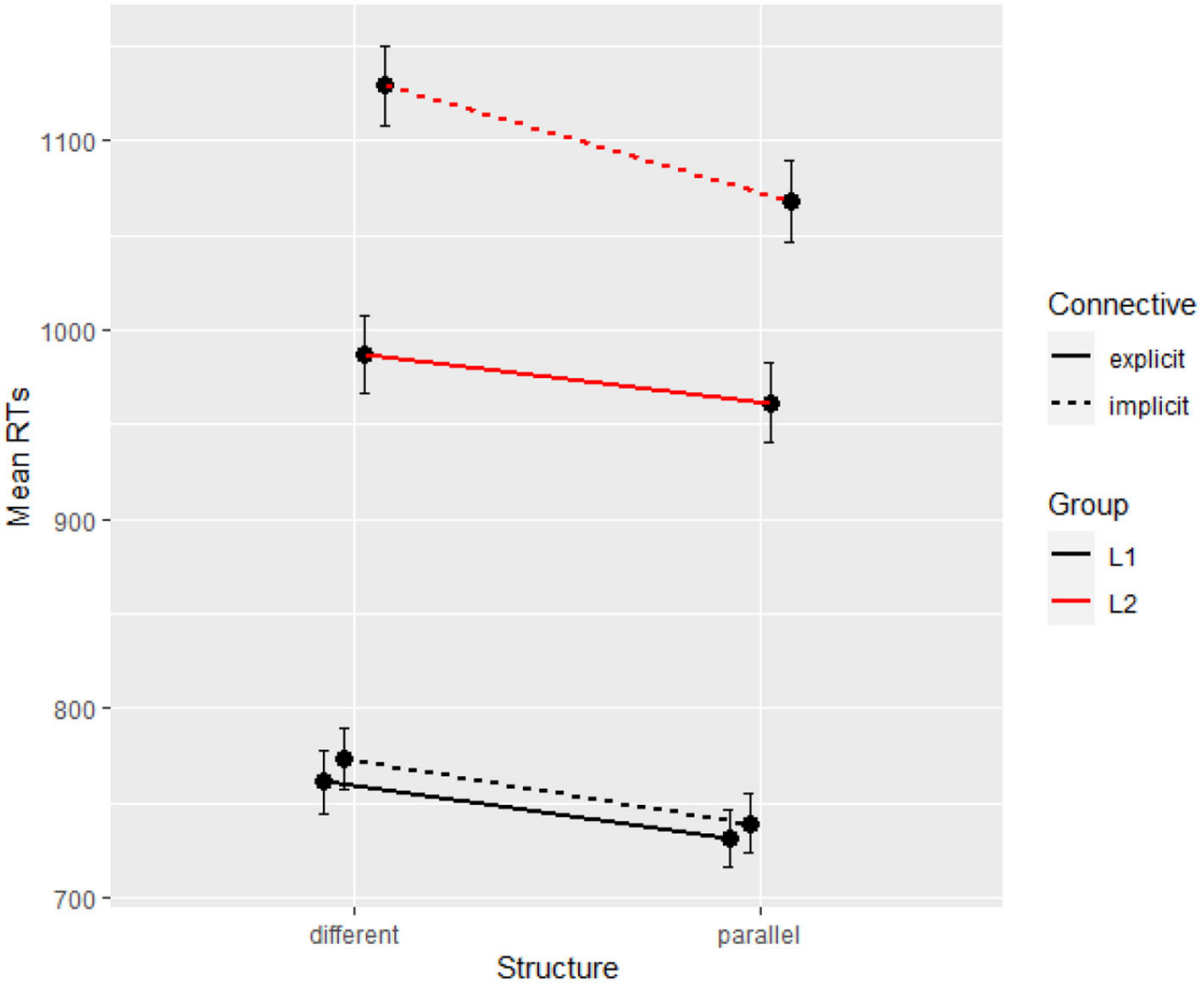
Mean reading times per condition and per group (Experiment 1).

Figure 1 reports conditional means for both groups and shows a very large (200 ms) difference between groups.

### Discussion

The results of Experiment 1 show that native speakers can process contrastive relations as easily with or without a connective, but that they benefit from parallelism. By contrast, non-native speakers do benefit from a connective, with a 100 ms difference between explicit and implicit trials. This facilitation of the connective in the L2 group seems to trump the effect of parallelism, which can only be observed in implicit relations, where participants compensate for the absence of a lexical instruction (i.e., the connective) by using the information provided structurally (i.e., the parallelism). The very large difference between L1 and L2 participants confirms that ease of processing increases with language proficiency. In addition to this baseline difference, our data shows a different pattern related to the effect of lexical and structural cues: L2 readers tend to rely on connectives first and other contextual cues second, whereas, for L1 readers, connectives are more optional, yet indirect cues like parallelism can be beneficial.

The optionality of contrastive connectives in L1 may be surprising given that prior studies have consistently reported a facilitation effect of connectives (e.g., Sanders and Noordman, 2000 on problem-solution and list relations). However, it should be noted that contrast is rarely included in discourse processing studies, which tend to focus on other types of relations such as concession or result. As Crible (2020) observed in corpus data, contrastive relations (along with elaboration relations) tend to be expressed by contextual cues more often than other relations, which means that the instruction of the connective might be redundant with the information already present in the segments themselves. As mentioned before, contrast is necessarily marked semantically, which makes it highly accessible to native speakers.

Overall, Experiment 1 confirmed the facilitation effect of parallelism in contrastive relations, except in L2 when the relation is marked by the explicit connective *par contre*, in which case the connective is informative enough. This result raises the question of what would happen if non-native speakers are confronted with a less familiar connective. We can expect that they will have a lower ability to use its information, and hence may rely more on parallelism in such cases. We assess this possibility in Experiment 2.

## Experiment 2

### Predictions

This experiment tests the effect of connective frequency on processing and its interaction with parallelism across speaker groups (L1 and L2). We compare the connective *par contre* used in Experiment 1 with a synonymous but less frequent connective that is more restricted to writing, viz. *en revanche*. Previous studies (Zufferey et al., 2018) found immediate processing delays after connectives typical of the written register (French *car* “since”) compared to more spoken-like equivalents (*parce que* “because”) in native speakers. As a result, we expect shorter reading times for the more frequent connective *par contre* than for its more formal equivalent *en revanche* at the connective region (Segment 4), with possible spill-over on the verb phrase region (Segment 5) for both groups. In addition, offline tasks indicate that L2 readers do not master *en revanche* as well as *par contre* (Wetzel et al., 2020), which would result in a larger and more persistent effect for L2 than L1 readers. We further expect parallelism to reduce reading times for both groups at the verb phrase (Segment 5) and critical region (Segment 6), particularly with the less frequent connective *en revanche* as a compensation strategy, similarly to the L2 pattern found for implicit relations in Experiment 1. We once more expect a strong group difference as an effect of proficiency.

### Participants

For this experiment, 80 native and 80 non-native speakers of French who did not take part in Experiment 1 were recruited on Prolific according to the same pre-screening criteria (55% female, 33yo on average, aged 18–73yo) and were remunerated £2.5 for their contribution. All participants gave informed consent before participation.

### Materials and Procedure

The items and fillers were the same as in Experiment 1, except that we replaced the implicit condition with a second explicit condition with the connective *en revanche*. This connective was chosen because it is less frequent and bound to the written mode, which is likely to make it more difficult to understand for non-native speakers. Indeed, L2-learners of French struggled more to insert *en revanche* in a sentence-cloze task than the more common *par contre* (Wetzel et al., 2020).

This results in a new 2 × 2 design where each connective (frequent *par contre* vs. infrequent *en revanche*) combines with each structure (parallel or not). A second connective was also introduced to replace the implicit condition for fillers: *du coup* (*so*) vs. *ainsi* (*therefore*) for result, *parce que* (*because*) vs. *car* (*for*) for cause and *et … aussi* (*and…similarly*) vs. *de même* (*likewise*) for similarity. These pairs of filler connectives were chosen because they mirror the difference in register found between *par contre* and *en revanche*, following Wetzel et al. (2020). Because all items are presented with an explicit connective, we modified the question: a comprehension question targeting the type of discourse relation would have been too easy and could have encouraged participants to focus only on the connective segment. As a result, we decided to replace it with a verification statement that targeted anything in either the first or second segment of the item. Participants had to indicate whether the statement was true or false.

The rest of the procedure is the same as in Experiment 1. Participants took about 29 min to complete the study on average.

### Results

The 80 L1 participants reached an overall accuracy of 94.23% on the verification question in this experiment (range 73–99), which shows that they were reading for understanding. The 80 L2 participants reached 88.45% overall accuracy (42–99). We discarded one L2 participant who scored below 50% for contrastive items. We then removed extreme values (41 cases) and replaced outliers following the same method as in the previous experiment (segment 4: 224; segment 5: 201; segment 6: 194, around 3% of the data each).

**Figure 2:**
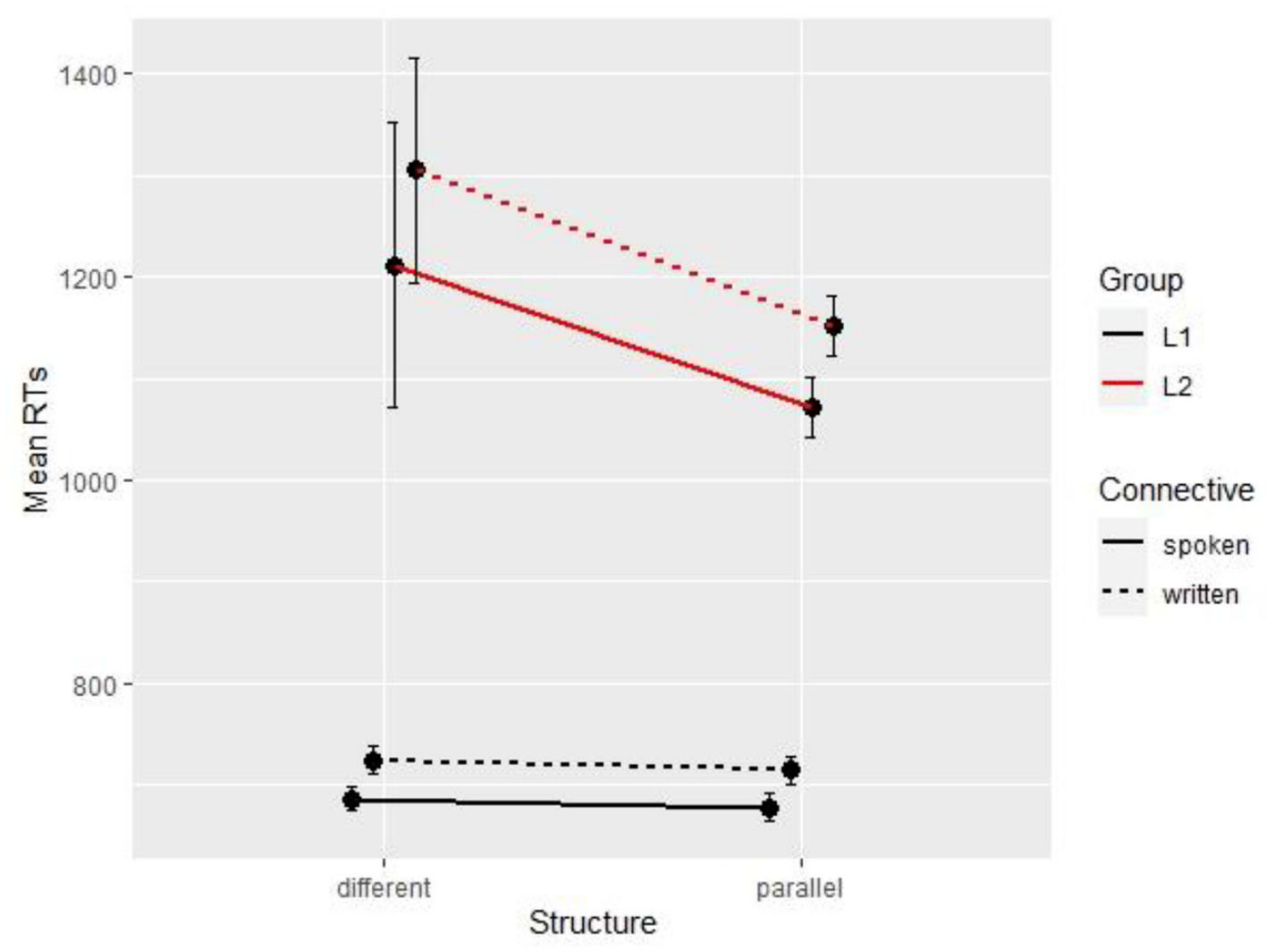
Mean reading times per condition and per group (Experiment 2, segment 4).

Starting with the connective region (segment 4), in the L1 data, the base model with random intercepts by participant and by item was not improved by Structure (Δχ^2^ = 1.08, Δ*df* = 1, *p* = 0.3) but was significantly improved by Connective (Δχ^2^ = 17.34, Δ*df* = 1, *p* < 0.001). The final model returns a main effect of connective with longer reading times for the written connective *en revanche* (*M* = 720 ms; *SD* = 396) than for the more frequent *par contre* (*M* = 682 ms; *SD* = 371). Similarly, in L2, the base model with random intercepts by participant and by item was not improved by Structure (Δχ^2^ = 0.06, Δ*df* = 1, *p* = 0.81) but was significantly improved by Connective (Δχ^2^ = 24.18, Δ*df* = 1, *p* < 0.001). The final model returns a main effect of connective with longer reading times for the written connective *en revanche* (*M* = 1.228 ms; *SD* = 2,280) than for the more frequent *par contre* (*M* = 1.142 ms; *SD* = 2,843). Figure 2 shows the means for all conditions and groups on the connective region.

The connective effect carries over to segment 5 (verb phrase region) for native readers only, with slightly longer reading times for *en revanche* (*M* = 627 ms; *SD* = 301) than *par contre* (*M* = 612 ms; *SD* = 329). The regression coefficients for all segments with significant effects are reported in Tables 5, 6 for L1 and L2 readers, respectively.

**Table 5:** Model of the L1 data on segments 4 and 5 (Experiment 2).

	**β**	***SE***	***t***	***p***
Segment 4				
Written connective	0.05	0.01	4.17	^***^
Segment 5				
Written connective	0.04	0.01	2.8	^**^
Parallel structure	−0.03	0.01	−1.9	

** *p < 0.01 and*

*** *p < 0.001*.

**Table 6:** Model of the L2 data on segments 4, 5, and 6 (Experiment 2).

	**β**	***SE***	***t***	***p***
Segment 4				
Written connective	0.07	0.02	4.92	^***^
Segment 5				
Parallel structure	−0.12	0.01	−9–12	^***^
Segment 6				
Parallel structure	−0.03	0.01	−2.26	^*^

* *p < 0.05 and*

*** *p < 0.001*.

Moving on to the critical segment (final region), in L1, the base model with random intercepts by participant and by item was not significantly improved by any predictor (Structure: Δχ^2^ = 2.55, Δ*df* = 1, *p* = 0.11; Connective: Δχ^2^ = 0.01, Δ*df* = 1, *p* = 0.97), which indicates that the difference between connectives was no longer true at the end of the sentence and that native readers found all conditions equally easy to process at that stage (around 685 ms on average).

In L2, the model with random intercept per participant and item was improved by Structure (Δχ^2^ = 5.1, Δ*df* = 1, *p* < 0.05) but not by Connective (Δχ^2^ = 1.96, Δ*df* = 2, *p* = 0.38). The final model returns a main effect of parallelism with shorter reading times in parallel (*M* = 1,147 ms; *SD* = 762) than non-parallel trials (*M* = 1,191 ms; *SD* = 780).

**Figure 3:**
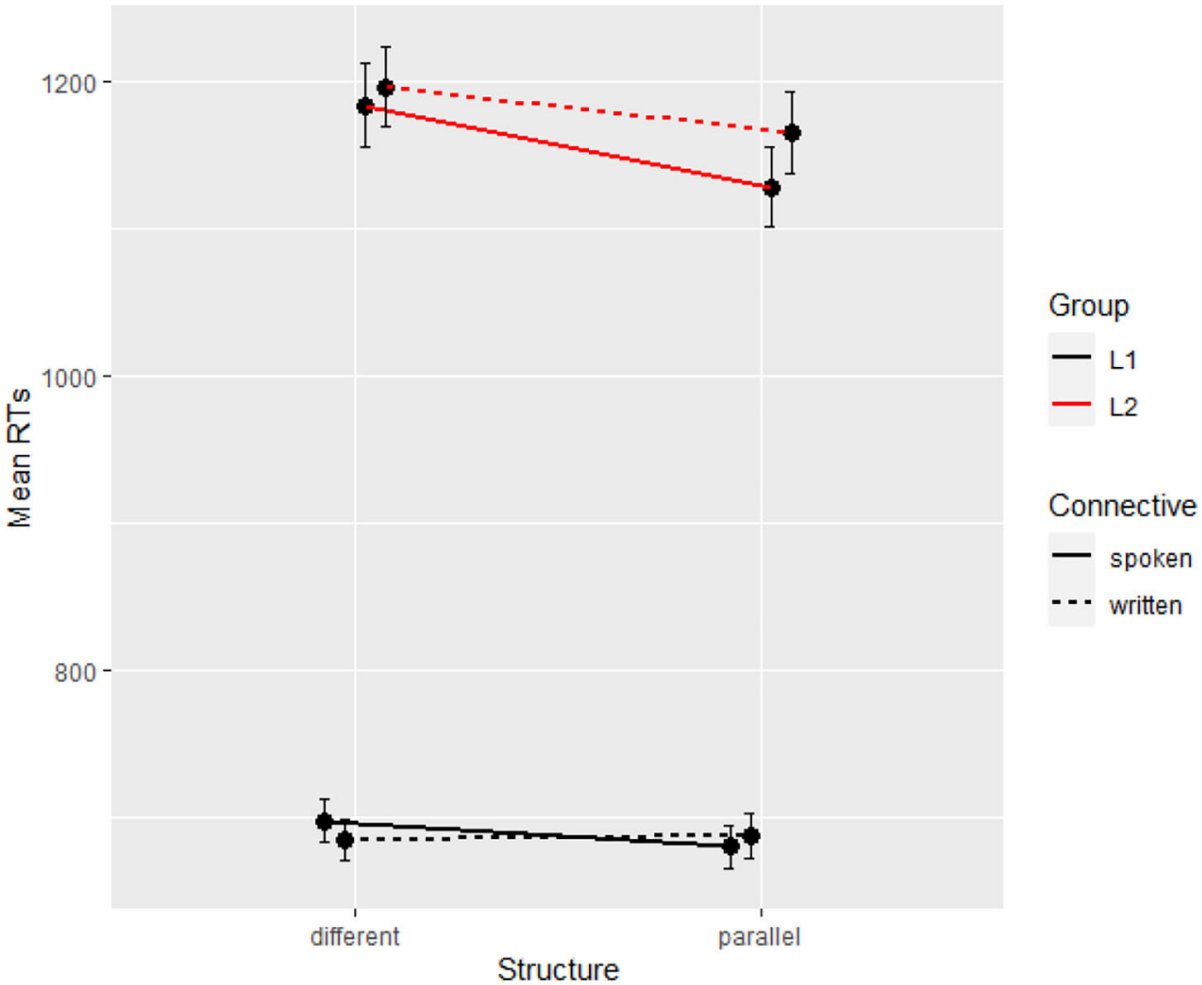
Mean reading times per condition and per group (Experiment 2, segment 6).

L1 participants scored 44.34 on the Lextale test (range 12–56) against 25.59 for L2 participants (0–51), which is a highly significant difference (ß = −0.33, *SE* = 0.03, *t* = −12.39, *p* < 0.001) that confirms the participants' own assessment. With both groups combined, in the final segment, the base model on reading times with random intercepts per participant and per item was improved by Structure (Δχ^2^ = 7.57, Δ*df* = 1, *p* < 0.01) and by Group (Δχ^2^ = 52.33, Δ*df* = 2, *p* < 0.001), not by Connective (Δχ^2^ = 1.70, Δ*df* = 2, *p* = 0.43). The final model returns only a main effect of group with longer RTs in L2, suggesting that the effect of group is so large that it erases that of parallelism which was otherwise significant in that segment in the L2 data (coefficients in Table 7). Figure 3 reports the means for all conditions and groups on the critical segment, and shows again a very large (400 ms) group difference.

**Table 7:** Model of both L1 + L2 data on segment 6 (Experiment 2).

	**β**	***SE***	***t***	***p***
L2 Group	0.51	0.06	7.85	^***^

*** *p < 0.001*.

### Discussion

Experiment 2 confirmed the early slow-down effect of high-register connectives, with both groups of participants processing *par contre* faster than *en revanche* at the connective region, and thus supports the findings in an offline-task by Wetzel et al. (2020). However, this effect disappeared by the end of the sentence for both groups, which shows that the initial difficulty of *en revanche* was soon resolved. This finding is in line with previous studies on connective processing (e.g., Canestrelli et al., 2013; Zufferey et al., 2018). As a result, we did not find the expected interaction between parallelism and connectives in the final segment, which suggests that the frequency or register of the connective does not create enough of a lasting difficulty for parallelism to act as a compensation cue.

Nevertheless, the effect of parallelism was significant for the L2 group in the final segment, contrary to Experiment 1, which matches our predictions for reading parallel structures in a second language. Due to the repetition of an element which is already decoded, the processing of the sentence is facilitated and coherence relations are interpreted faster. This effect likely reflects the basic comprehension priming effect of parallelism, whereby linguistic materials that have already been processed are easier to process the second time (e.g., Frazier et al., 1984). Due to its great accessibility, this effect is especially beneficial when reading in a second language. These findings thus suggest that parallelism appears indeed to be an informative and accessible cue, helping L2 readers identify the underlying coherence relation. As predicted, L2 readers benefited from parallel structures in this experiment even to a higher extent than native speakers.

This facilitation is not specific to contrastive relations, however, and does not inform us of the way L2 readers process different discourse cues. What the results show instead is that, while they are highly sensitive to the presence vs. absence of a connective (cf. Experiment 1), the impact of the type of connective (its frequency) is short-lived, as far as contrastive relations are concerned. In the next experiment, we further explore this issue by comparing an appropriate contrastive connective (*mais* “but”) with an ambiguous connective that can be used in contrast but needs to be pragmatically enriched by inference in order to be fully interpreted (*et* “and”). This shall refine our scale of sensitivity to lexical and structural cues for L1 and L2 readers.

## Experiment 3

### Predictions

In the final experiment, we replaced *par contre* and *en revanche* with the conjunctions *mais* “but” and *et* “and,” which differ in their semantics and the resulting pragmatic processes that are involved to interpret them. While *mais* encodes contrast, *et* is semantically additive, so that using *et* in a contrastive relation requires the comprehender to infer a non-literal use of the conjunction. This mismatch between the semantics of the connective and its pragmatic interpretation only becomes apparent in the final segment. As a result, we expect that reading times of the final segment will be longer with *et* than *mais* and that the facilitation of parallelism will be stronger with the former, to compensate for the ambiguity of the connective. We will also explore reading times of the connective and verb phrase regions in order to test whether the ambiguity of *et* has an immediate effect or only emerges later. This design is inspired by Crible and Pickering's (2020) Experiment 3 and extends it to L1 and L2 participants. We thus expect to replicate their pattern of findings with native French speakers. Besides a baseline group difference, it is unclear how L2 readers will process the ambiguity of *et* in interaction with parallel structures.

### Participants

For this experiment, 80 native and 80 non-native speakers of French who did not take part in the previous two experiments were recruited on *Prolific* according to the same pre-screening criteria (61% female, 32yo on average, aged 18–66yo) and were remunerated £2.5 for their contribution. All participants gave informed consent before participation.

### Materials and Procedure

The items and fillers were the same as in Experiments 1 and 2, except that we changed the connectives for contrastive items to *mais* (“but”) and *et* (“and”). Connectives in the filler items were also changed for result and similarity relations (*donc* “so” vs. *et* “and,” *de même* “similarly” vs. *et* “and,” respectively) in order to better match the contrastive conditions and to introduce *et* in three different relations, together with a more specific connective (*mais, donc, de même*); *et* is not possible in causal relations so we re-used the same pair as in Experiment 2 (*parce que* vs. *car*). This allowed us to avoid any one-to-one association between *et* and a specific relation, and to re-use the discourse-focused comprehension task from Experiment 1. Participants took about 26 min to complete the study on average.

### Results

The L1 participants reached an overall accuracy of 87.5% on the comprehension question in this experiment (range 37–99), with one participant below 50% on contrastive trials, who was therefore discarded. The L2 group reached 77.94% overall accuracy (20–98), from which we discarded eight participants below 50% for contrastive items. Extreme values (64) and outliers (segment 4: 200; segment 5: 178; segment 6: 203; around 3% of the data each) were dealt with following the same procedure as before.

**Figure 4:**
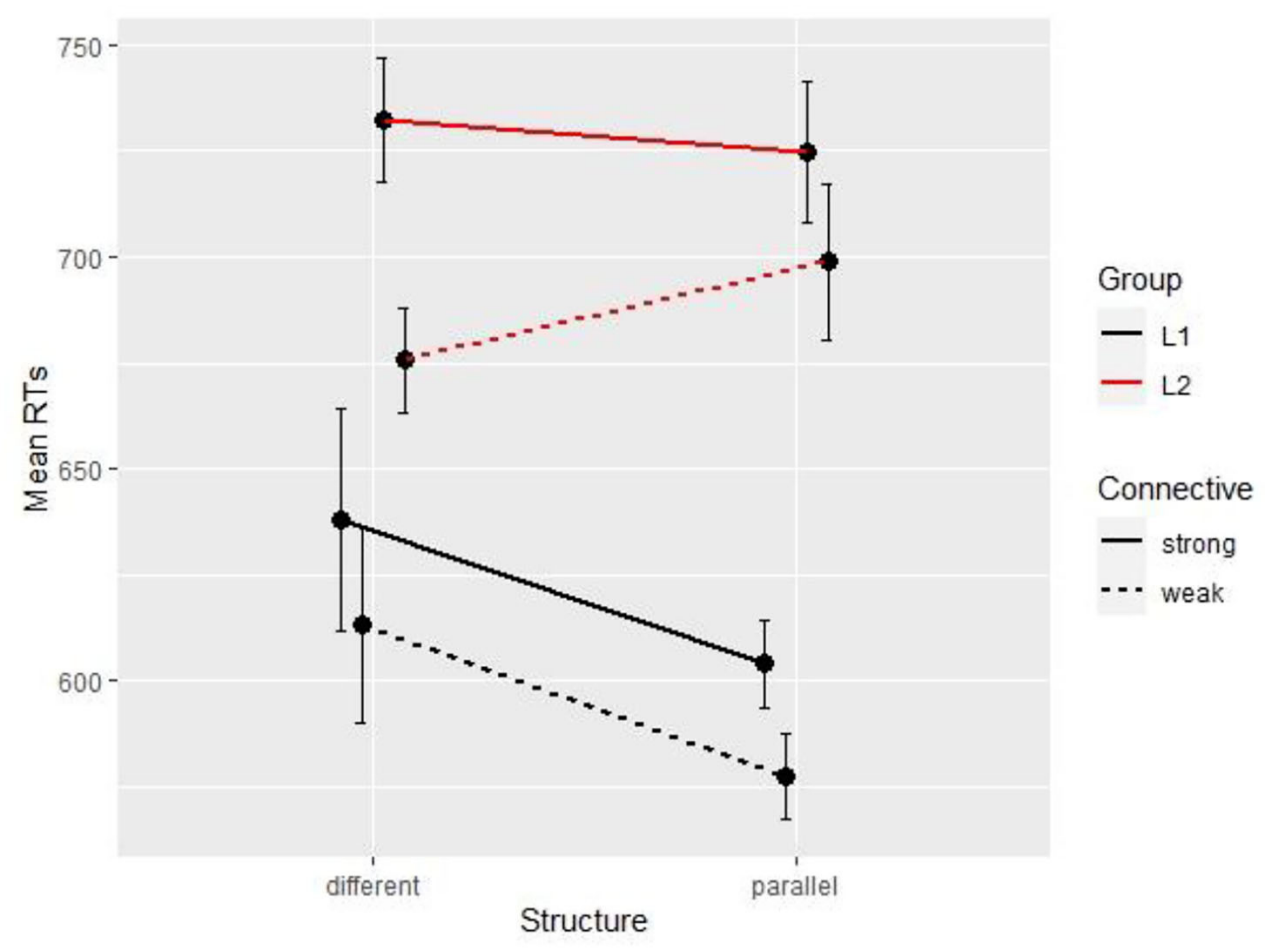
Mean reading times per condition and per group (Experiment 3, Segment 4).

At the connective region, in L1, the base model with random intercepts by participant and by item was not improved by Structure (Δχ^2^ = 1.01, Δ*df* = 1, *p* = 0.31) but was significantly improved by Connective (Δχ^2^ = 14, Δ*df* = 1, *p* < 0.001). The final model returns a main effect of Connective with shorter reading times with *et* (*M* = 595 ms; *SD* = 498) than *mais* (*M* = 621 ms; *SD* = 559). Similarly, in L2, the base model with random intercepts by participant and by item was not improved by Structure (Δχ^2^ = 0.1, Δ*df* = 1, *p* = 0.75) but was significantly improved by Connective (Δχ^2^ = 28.38, Δ*df* = 1, *p* < 0.001). The final model in L2 returns a main effect of Connective (ß = −0.06, *SE* = 0.01, *t* = −5.34, *p* < 0.001) with shorter reading times with *et* (*M* = 687 ms; *SD* = 416) than *mais* (*M* = 729 ms; *SD* = 419), as shown on Figure 4.

**Figure 5:**
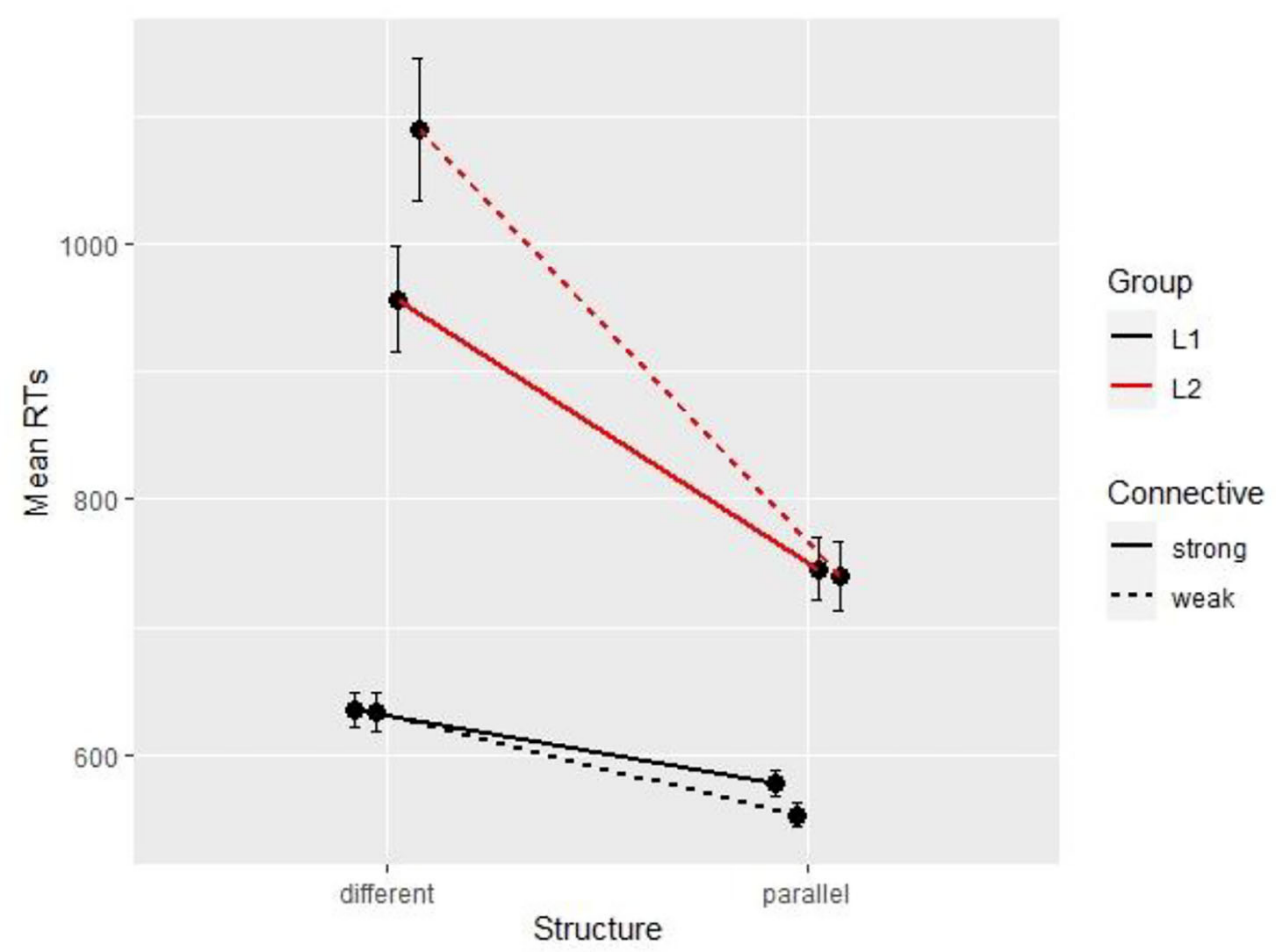
Mean reading times per condition and per group (Experiment 3, Segment 5).

The effect of connective does not carry over to the verb phrase region in L1, with only a facilitation effect of parallelism. However, in L2, the base model with random intercepts by participant and by item is improved by both Structure (Δχ^2^ = 265, Δ*df* = 1, *p* < 0.001) and Connective (Δχ^2^ = 9.02, Δ*df* = 2, *p* < 0.05). The final model on segment 5 returns a main effect of parallelism, of connective and a significant interaction, such that sentences with *et* are read more slowly than *mais* in non-parallel conditions, while there is no difference between connectives when combined with parallelism, as shown on Figure 5.

The regression coefficients for all segments with significant effects are reported in Tables 8, 9 for L1 and L2 readers, respectively.

**Table 8:** Model of the L1 data on segments 4, 5, and 6 (Experiment 3).

	**β**	***SE***	***t***	***p***
Segment 4				
Ambiguous connective	−0.04	0.01	−3.75	^***^
Segment 5				
Parallel structure	−0.07	0.01	−4.64	^***^
Segment 6				
Ambiguous connective	0.05	0.02	2.3	^**^
Connective ^*^ Structure	−0.04	0.02	−1.81	

* *p < 0.05*,

** *p < 0.01, and*

*** *p < 0.001*.

**Table 9:** Model of the L2 data on segments 4, 5, and 6 (Experiment 3).

	**β**	***SE***	***t***	***p***
Segment 4				
Ambiguous connective	−0.06	0.01	−5.34	^***^
Segment 5				
Parallel structure	−0.18	0.02	−9.79	^***^
Ambiguous connective	0.05	0.02	2.62	^**^
Connective * Structure	−0.08	0.03	−2.9	^**^
Segment 6				
Parallel structure	−0.06	0.02	−3.03	^**^
Ambiguous connective	0.08	0.02	4.35	^***^

** *p < 0.01 and*

*** *p < 0.001*.

Finally, on the critical segment, in L1, the base model with random intercepts by participant and by item was significantly improved by Structure (Δχ^2^ = 8.55, Δ*df* = 1, *p* < 0.01) and by Connective (Δχ^2^ = 9.15, Δ*df* = 2, *p* < 0.05). The final model returns a main effect of Connective with longer reading times with *et* (*M* = 774 ms; *SD* = 420) than *mais* (*M* = 761 ms; *SD* = 452), and a marginally significant interaction between factors. Pairwise comparison shows significant differences between non-parallel *et* and non-parallel *mais* (*p* < 0.05), between parallel *et* and non-parallel *et* (*p* < 0.01) and between parallel *mais* and non-parallel *et* (*p* < 0.001), which are the two most opposite conditions.

In L2, the model on the final segment with random intercept per participant and item was improved by Structure (Δχ^2^ = 9.89, Δ*df* = 1, *p* < 0.01) and by Connective (Δχ^2^ = 53.81, Δ*df* = 2, *p* < 0.001). The final model returns a main effect of parallelism with shorter reading times in parallel (*M* = 1,021 ms; *SD* = 597) than non-parallel trials (*M* = 1,069 ms; *SD* = 643). It further returns a main effect of connective with *et* being read more slowly (*M* = 1,096 ms; *SD* = 644) than *mais* (*M* = 994 ms; *SD* = 593).

Native participants scored 43.19 on the Lextale test (range 18–56) against 15.25 for non-native participants (−34–54), which is a highly significant difference (ß = −0.31, *SE* = 0.03, *t* = −11.54, *p* < 0.001). With both groups combined, the base model on reading times with random intercepts per participant and per item was improved by Structure (Δχ^2^ = 18.37, Δ*df* = 1, *p* < 0.001), by Connective (Δχ^2^ = 45.62, Δ*df* = 2, *p* < 0.001) and by Group (Δχ^2^ = 53.49, Δ*df* = 4, *p* < 0.001). The final model returns a main effect of group with longer RTs in L2, a main effect of Connective, a significant three-way interaction between all predictors and marginally significant interactions between Structure and Connective and between Structure and Group (coefficients in Table 10).

**Table 10:** Model of both L1 + L2 data on segment 6 (Experiment 3).

	**β**	***SE***	***t***	***p***
L2 Group	0.3	0.05	5.76	^***^
Ambiguous connective	0.05	0.02	2.89	^**^
Structure ^*^ Connective	−0.04	0.03	−1.77	
Structure ^*^ Group	−0.05	0.03	−1.78	
Structure ^*^ Connective ^*^ Group	0.08	0.04	2.07	^*^

* *p < 0.05*,

** *p < 0.01, and*

*** *p < 0.001*.

**Figure 6:**
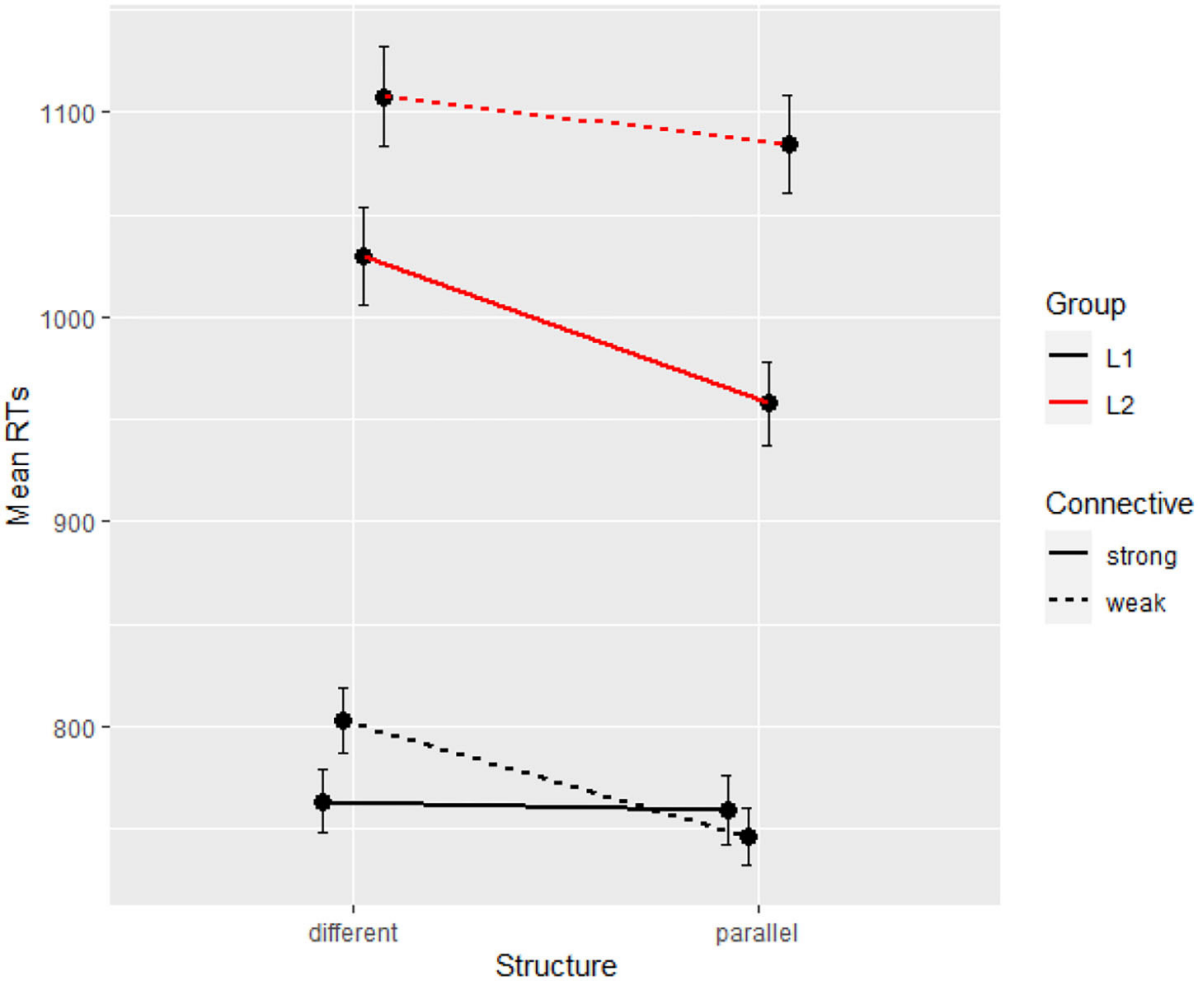
Mean reading times per condition and per group (Experiment 3, Segment 6).

Figure 6 shows the conditional means for all conditions and groups on the critical segment, where we can see that the effect of parallelism is in the expected direction for L1 readers (i.e., larger with the weak connective *et* than the stronger option *mais*) but in the opposite direction for L2 readers, with no difference between parallel and non-parallel trials with *et*.

### Discussion

This experiment introduced another factor of complexity in discourse processing, namely inferential processes for ambiguous connectives, which seems to have a different impact over time and across L1 and L2 readers. In L1, there is an early difference between connectives, with faster reading times for *et* than *mais*, which might reflect their difference in informativity (more information to process in *mais* than *et*) and an initial underspecified processing of *et*. The following segment is only affected by parallelism. In the final segment, the effect of connective re-appears in the reverse yet expected direction, with longer reading times for *et* once the contrastive relation is identified. These results on the critical segment replicated the pattern found by Crible and Pickering (2020), with a facilitation effect of parallelism that only comes into play with the ambiguous *et* as a compensation cue. This exact replication of their study on English suggests that parallelism is equally accessible to English and French native speakers.

In L2, the connective region reflects the underspecified processing of *et* compared to the immediate delay associated with *mais*, similarly to native readers. At the verb phrase region, the difference between connectives is restricted to non-parallel items and shows the opposite direction of effect (i.e., *et* longer than *mais*), which might already reflect the effort associated with the incremental enrichment of the relation without a strong connective. In the final segment, the effect of parallelism no longer applies in sentences containing *et* but does facilitate the processing of sentences with *mais*, contrary to the interaction found in the L1 data. In the case of non-native readers, the effect of parallelism is thus not restricted to ambiguous connectives. At the end of the sentence, *et* remains much harder to process than *mais*, while the difference between connectives is smaller in L1 and only significant in the non-parallel condition. The L2 data therefore shows that ambiguous connectives are much more problematic for non-native than native readers and cannot be compensated for by structural cues, unlike what we observed for implicit relations in Experiment 1. This suggests that implicitness and ambiguity have different effects on L2 discourse processing: pragmatic enrichment without any lexical cue is facilitated by parallelism, while it remains difficult in the presence of an ambiguous lexical cue, with or without parallelism. This could reflect the tendency of non-native readers to rely more strongly on the literal additive meaning of *et* at first, which hinders the derivation of a contrastive meaning through a process of inference. As a result, a structural cue for contrast did not help or hinder their processing of the relation, because this contrastive relation was not immediately available to them. In other words, the benefit of a structural cue like parallelism is canceled if it is not apparently convergent with the literal instruction of the lexical cue, which once again shows that connectives prevail over other cues for L2 readers (cf. Experiment 1). However, this conclusion is only tentative, as other factors may have played a role, such as the experimental settings where learners might feel pressured to strictly follow the semantic encoding.

### General Discussion

We conducted three self-paced reading experiments to determine how parallelism interacts with different types of connectives to help native and non-native readers process contrastive relations. We manipulated the presence, frequency and ambiguity of the connective and observed different facilitation effects across speaker groups and across tasks.

For L1 speakers, the effect of parallelism seems to be modulated by task difficulty: when the task is easy, structural or lexical cues don't make a difference in processing ease because reading is shallow and automatic (absence of effect in Experiment 2 with the simple verification task); when the task is harder and always includes a connective, readers only benefit from parallelism when the relation is conveyed by the ambiguous connective (i.e., “and”), as the more informative connective (“but”) already gives sufficient information on the intended relation (Experiment 3); finally, with the same difficult task and the inclusion of implicit trials, readers pay more attention to contextual cues and benefit from parallelism across the board (Experiment 1). This suggests a cline from low complexity (no effect of parallelism) to intermediate (interaction effect) and high complexity (main effect of parallelism). In other words, the harder the task, the more prominent the facilitation effect of parallelism for L1 readers. This result complements Crible and Pickering's (2020) findings by showing the specific conditions in which the combination of lexical and structural cues is beneficial to readers.

For L2 readers, on the other hand, the effect of parallelism is more pervasive. It was observed in the easy task of Experiment 2, in the harder task of Experiment 3 with *mais* and in the same harder task with implicit relations. All these contexts can be considered as cognitively demanding for non-native readers, considering their lower level of linguistic proficiency: the association between parallelism and task difficulty therefore holds for L2 readers as well, with the difference that, for them, all tasks were difficult. The complexity factor could also explain why we did not find an effect of parallelism in explicit relations in Experiment 1 in this population: the explicit condition was comparatively much easier than the implicit one regardless of parallelism, and we suggested that in such contexts, the presence of a connective trumps any other cue. Finally, parallelism had no effect when the contrastive relation was marked by the underspecified connective *et* (“and”). We argued that this can be explained by the prevalence of its literal meaning of addition for non-native readers, thus canceling any reinforced inference from parallelism, although this is only a tentative suggestion at this stage.

Overall, our study shows that parallelism has a larger effect on non-native than native readers and, crucially, that this effect is subject to different factors across the two groups: in L2, its facilitation effect is smaller than that of an explicit connective, and is only recruited with literal uses of connectives. By contrast, in L1 its effect seems largely explained by task difficulty, understood here as both the nature of the task (comprehension vs. verification) and the explicitness of the lexical cue. In other words, native readers only need reinforcing cues in more complex tasks, whereas non-native readers benefit from all types of cues to perform any task, provided that these signals are clear and accessible. In this respect, our results are fully coherent with those of Crible and Pickering (2020), and therefore provide further confirmation for the role of parallelism in discourse processing. In addition, they raise the issue of task difficulty and the need to include several different tasks in reading studies in order to better understand the conditions that increase processing complexity for readers.

One limitation of this study relates to our pool of participants. Web-based experiments typically include more varied participant profiles than laboratory experiments, which usually recruit from restricted populations such as university students. Still, this intrinsic variation is not too problematic for psycholinguistic studies (Enochson and Culbertson, 2015) and can be managed by larger samples. However, in our data, we further noticed that the L2 participants from Experiment 3 had a significantly lower mean score on the Lextale test (*M* = 15.25) compared to the first two experiments (*M* = 23.69 and 25.59), [*F*_(2, 4486.3)_ = 10.83, *p* < 0.001]. While this was beyond our control, as participants self-register for studies on Prolific, it may have influenced the results, in particular those concerning the processing of ambiguous *et*: it might be the case that the non-literal contrastive use of *et* would have been accessible to higher-proficiency bilinguals, in which case the effect of parallelism would have applied to this connective as well, as it did for implicit relations in Experiment 1. This observation suggests that language proficiency is indeed a crucial factor in reading behaviors and processing difficulty at the discourse level and beyond (e.g., Zufferey and Gygax, 2020), which calls for more studies integrating participants with different language backgrounds. Further factors of individual variation such as exposure to print (Zufferey and Gygax, 2020) or type of language input (e.g., classroom vs. naturalistic, cf. Gilquin, 2016) would also complement the complex picture of interacting factors that the present study has strived to sketch. We leave this for future research.

Taken together, our three experiments convincingly showed that a structural feature of the segments, namely parallelism, helped native and non-native speakers in their online processing of contrastive relations. This facilitation is modulated by task complexity for L1 readers, while it is more pervasive for L2 readers but subject to other factors, including the availability of non-literal interpretations. To our knowledge, this is the first study that examined the effect of non-connective cues in L2 discourse processing. As such, it opens up many perspectives for further research, including on the role of different types of cognitive load manipulations or possible transfer effects, which should also be extended to a broader range of discourse relations.

## Data Availability Statement

The datasets presented in this study can be found on the online repository at: https://osf.io/mwy7q/?view_only=57847a6801e04bdead0a51f1bab97a0f.

## Ethics Statement

The studies involving human participants were reviewed and approved by SNSF: Division I. The patients/participants provided their written informed consent to participate in this study. Written informed consent was obtained from the individual(s) for the publication of any potentially identifiable images or data included in this article.

## Author Contributions

LC was the lead investigator in this study, she took care of material preparation, programming and running the experiment, data analysis, and writing up the paper. MW and SZ were both involved at all stages of the study and contributed to all decisions, piloting the experiment, interpreting the data and revising the manuscript, and were particularly responsible for all aspects related to non-native processing. All authors contributed to the article and approved the submitted version.

## Conflict of Interest

The authors declare that the research was conducted in the absence of any commercial or financial relationships that could be construed as a potential conflict of interest.

## Footnotes

^1^The mean score and range on the Lextale test for the L2 group is similar to Wetzel's et al. (2020) study where they tested a more homogeneous group of participants with a B1–B2 proficiency level [M = 14.69; SD = 8.37; range (−3–37)].
