# Supplementary figures and images for: Lexical and Structural Cues to Discourse Processing in First and Second Language
Source: Front Psychol. 2021 Jul 1;12:685491. doi: 10.3389/fpsyg.2021.685491 (PMC8280496; doi:10.3389/fpsyg.2021.685491)

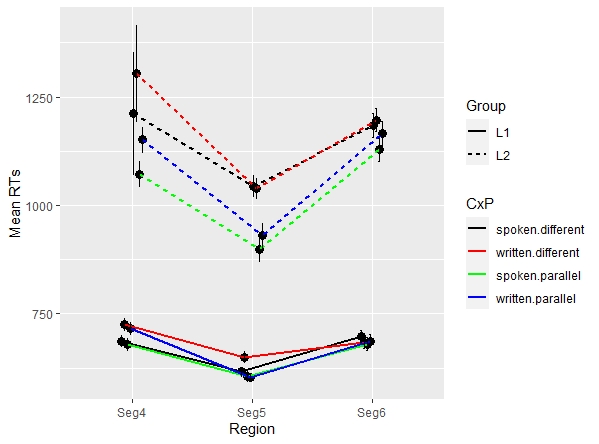

Supplement: Supplementary file 1 [file Image_1.JPEG]

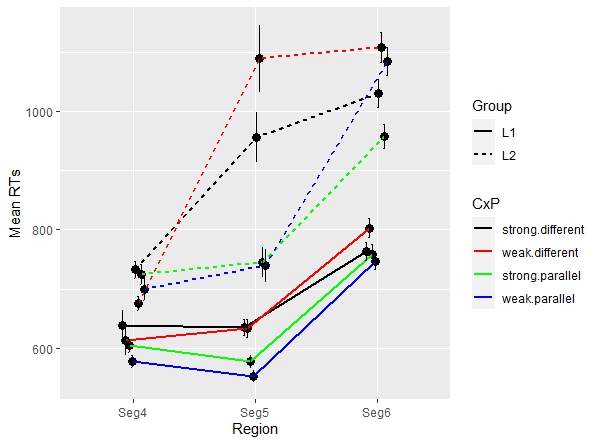

Supplement: Supplementary file 2 [file Image_2.JPEG]
